# Monocyte-derived dendritic cells can be detected in urine of kidney transplant recipients with pathogenic asymptomatic bacteriuria

**DOI:** 10.3389/frtra.2024.1366104

**Published:** 2024-06-12

**Authors:** Vanja Salvadé, Oriol Manuel, Dela Golshayan, Carolina Obregon

**Affiliations:** ^1^ Transplantation Centre, Departments of Medicine and Surgery, Lausanne University Hospital (CHUV), Lausanne, Switzerland; ^2^ Infectious Diseases Service, Department of Medicine, Lausanne University Hospital (CHUV) and University of Lausanne (UNIL), Lausanne, Switzerland; ^3^ Woman-Mother-Child Department, Pediatric Service, Pediatric Nephrology Laboratory, Centre Hospitalier Universitaire Vaudois, Lausanne, Switzerland

**Keywords:** UTI, asymptomatic bacteriuria, urine, kidney transplantation, dendritic cells, monocyte-derived dendritic cell

## Abstract

Urinary tract infections (UTI) are an important clinical problem in kidney transplant recipients (KTR). Asymptomatic bacteriuria (ASB) is frequent in these patients and often resolved by the immune system, but a significant proportion may progress to complicated UTI, which may compromise allograft function and survival. It is essential to determine the involvement of the immune system in the infectious process. Dendritic cells (DCs) are recognised as playing a pivotal role in initiating inflammatory responses capable of priming antigen-specific T cells, a crucial step in determining the fate of local inflammation. Little is known about their role in the control of UTI. In this brief communication, we report an incidental finding in a group of 16 stable KTR in which monocyte-derived dendritic cells (ModDCs), analysed by flow cytometry, were found in urine of patients with ASB and high bacterial counts >10^7^ cfu/ml. Within this group, one patient developed pyelonephritis in the following days. These findings suggest that the immune system, in particular DCs, may be recruited during the course of a UTI and, to our knowledge, present for the first time evidence that inflammatory ModDCs can be detected in urine. Their frequency may reflect the degree of infection. This finding suggests the potential for exploring whether these cells may be useful in distinguishing between pathogenic ASB and those that can be resolved by the immune system.

## Introduction

Asymptomatic bacteriuria (ASB) is frequent in kidney transplant recipients (KTR). It is thought to be a risk marker for developing a urinary tract infection (UTI) which, if not controlled in time, represents an adverse event for these patients (1). Several factors contribute to increased susceptibility to UTI in KTR, including the patient's genetic and immunological background, the anatomy of the native kidneys, the origin of the donor organ and surgical complications during transplantation (2, 3). Although the “net state of immunosuppression” has been postulated as a conceptual framework linking immunosuppression and risk of infection, studies measuring the actual impact of immunosuppression as a cause of increased UTI, and immune system involvement in the infectious process, are incomplete (4).

Bacteriuria is defined by the presence of >10^5^ bacterial colony forming units per millilitre (cfu/ml) in urine, which can be asymptomatic (ASB) or symptomatic, ranging from local symptoms, such as cystitis, to the development of pyelonephritis and sepsis, which in transplant recipients can have severe consequences including impaired allograft function, allograft loss and death. However, the correlation of UTI or even pyelonephritis as independent risk factors for graft rejection has been the subject of controversy. This issue can be largely explained due to study differences in the classification of clinical symptoms, antibiotic treatment protocols or categorization of events (5, 6).

Importantly, these studies mainly include microbiological and clinical data, but none of them evaluate the host immune response. In addition, clinical manifestations defined by symptoms, although useful, are not sufficient to exclude a harmful bacterial infection. This is problematic for kidney transplant patients as recent clinical trials have shown that routine screening and treatment of ASB with antibiotics does not significantly reduce the development of cystitis or pyelonephritis. One study even demonstrated that the group treated with antibiotics developed more episodes of bacteriuria due to antibiotic resistance (7–11). Undoubtedly, new tools need to be developed that distinguish patients with potentially harmless ASB from those evolving towards complicated UTI or pyelonephritis.

Dendritic cells (DCs) play an important role in initiating local inflammatory responses capable of activating antigen-specific T cells in the lymph nodes, a crucial step in determining the outcome of inflammatory and infectious processes (12). Human and murine bona-fide DCs have been classified into 3 groups, plasmacytoid DCs and two conventional myeloid DCs called cDC1 and cDC2. Their function in human systemic and local inflammation has been difficult to establish because of their low frequency. However, a particular subtype of monocytes with DC functions, called monocyte-derived DC (ModDC), has been identified *in vivo*, with a frequency 10–20 times higher than their DC counterparts in tissues (13–17). In humans, in spite of the fact that it has been difficult to properly identify ModDCs, a phenotype expressing CD14, CD1c, DC-SIGN and CD141 has been described in inflammatory tissues such as arthritic synovial fluid or in the bronchoalveolar lavage fluid of patients with sarcoidosis (13, 18). Little is known about human ModDCs during UTI. During a pathogenic bacterial infection in the mouse model, it was shown that bladder phagocytes mount a coordinated innate defence against bacterial infections in which monocyte-derived cells activate resident macrophages via TNF-α to produce CXCL2 to attract and activate neutrophils, which are essential for the early control of the infection (19, 20). However, uncontrolled uropathogenic bacteria ascended from the bladder to the kidneys, increasing the risk of developing pyelonephritis. As for the innate response, mononuclear phagocytes have been shown to be located in the renal tubulointerstitial space able to detect the ascending bacteria and activate neutrophils. It is currently unknown whether and how an adaptive immune response can be triggered at a later stage of infection involving the kidney parenchyma.

Therefore, based on these previous observations, we investigated whether ModDCs, detected in urine samples of KTR with ASB, could be a biomarker for complicated UTI or pyelonephritis.

In the context of developing a prospective study to characterize ModDCs in the urine of KTR, with the overall objective of determining whether these cells could be associated with renal allograft dysfunction and rejection, when analysing samples from stable recipients, ModDCs were identified in the urine of a subgroup of patients who had an ASB with the highest bacterial counts. The fact that one patient in this group developed pyelonephritis in the following days raised several questions as to whether the frequency of this phenotype in the urine may reflect the severity of infection and to what extent it may be useful in distinguishing pathogenic ASB from those that can be resolved by the immune system. These findings raise the possibility of assessing whether ModDCs present in urine samples could serve as biomarkers for pathogenic ASB and UTI in KTR, as well as investigating their role in both local and systemic immune responses triggered by these infections, and the potential implications for graft outcome.

## Material and methods

### Patients

Sixteen KTR constituted the group of patients included in the analysis. These patients are part of a control KTR group with stable graft function, without BK viremia nor allograft rejection at the time of their inclusion, in a more comprehensive ongoing observational study, investigating the role of urine ModDCs in graft outcome after kidney transplantation. Patient characteristics are summarised in Table 1. No statistical method was applied to determine sample size. Inclusion criteria was stable post-transplant patients followed at our Transplantation center, regardless of the time after kidney transplantation, and no specific exclusion criteria was applied, except for lack of complete clinical and microbiological data. Written consent from the patients was obtained in accordance with the Cantonal Ethics Committee of the Canton of Vaud Switzerland (CER-VD/ protocol 2018-01149). Microbiological assessment was based on established standard criteria at the hospital, and bacteriuria was defined as the presence of >10^5^ cfu/ml in urine. Colony counts ≤10^5^ cfu/ml were considered a negative result and the absence of counts was considered sterile.

**Table 1:** Baseline characteristics of patients grouped according to PCA analysis.

Variables	Group 1. Sterile (*n* = 5)	Group 2. Bacterial counts ≤10^5 ^cfu/ml (*n* = 5)	Group 3a bacterial counts >10^5 ^cfu/ml (*n* = 2)	Group 3b bacterial counts >10^7 ^cfu/ml (*n* = 4)	*p*
Age, median (IQR)	57 (51–78)	55 (49–65)	56 (53–59)	58 (48–75)	0.8^a^
Female (%)	1 (20%)	2 (40%)	1 (50%)	4 (100%)	0.99^c^
Days after transplantation, median (IQR)	9 (8–277)	187 (52–277)	110 (107–113)	1020 (55–2278)	0.34^a^
Leukocytes urine (x10^6^/L), mean (SEM)	22.4 (11.7)	100.2 (91.5)	1134.5 (831.5)	569.7 (333)	0.065^a^
Creatinine urine (µmol/L), mean (SEM)	4085 (1024)	6376 (1444)	7106 (2694)	6007 (2179)	0.632^a^
Total proteins urine (g/L) mean (SEM)	0.50 (0.29)	0.30 (0.08)	0.38 (0.28)	0.18 (0.09)	0.71^a^
Bacteriuria (>10^5 ^cfu/ml) (%)	0 (0)	0 (0)	2 (100)	4 (100)	0.69^c^
Asymptomatic bacteriuria (%)	0 (0)	0 (0)	2 (100)	4 (100)	0.69^c^
Number of colony-forming unit (cfu/ml), mean (SEM)	0.0E + 00 (0)	4.2E + 04 (2.4E + 04)	5.5E + 06 (4.5E + 06)	3.3E + 07 (2.3E + 07)	**<0.0001** ^b^
Antibiotic treatment due to bacterial infection (%)	0 (0)	0 (0)	0 (0)	3 (75)	0.22^c^

Clinical, microbiological and laboratory data. Baseline characteristics of patients including clinical, microbiological and laboratory data used for principal component analysis. IQR, Interquartile range; SEM, standard error of the mean.

A statistically significant value is shown in bold.

^a^ One way ANOVA test.

^b^ Kruskall-Wallis test.

^c^ Two-way ANOVA test.

### Urine samples

Urine samples were collected in 200 or 500 ml sterile wide-necked bottles, as part of routine urine diagnostic sampling during follow-up after kidney transplantation. The clinic took the volume needed for diagnostic analysis and the remainder was used for the study. In 3 of the patients, we had the opportunity to collect a 12-hour urine sample. Urine samples were analysed immediately after collection, centrifuged, washed and the pelleted cells counted with Neubauer hemocytometry. According to our observations and those of other groups, the total number of cells in urine is independent of the voided volume (21). For flow cytometry analysis 5 × 10^5^ cells were stained per condition, however, two samples below this concentration were included. Cells were analysed as previously reported (13).

### Flow cytometry

Briefly, cell suspensions were stained with LIVE/DEAD® Fixable Dead Cell Stain (molecular probes) followed by surface staining with the following Abs: anti-FITC-CD1c, APC-CD141, PE-DC-SIGN, PerCP/Cy5.5-CD14, A700-CD11b, KO-CD16, PE/Cy7-HLA-DR, PB-CD56, PB-CD19, PB-CD3, PB-CD66b or isotype matched control antibodies (detailed information of the antibodies is shown in Supplementary Material Table S2). In brief, cells were stained for 20 min at 4 °C in FACS buffer and fixed in 1% paraformaldehyde. Cell-surface fluorescence intensity was assessed on a Gallios flow cytometer (Beckman Coulter) and analysed using FlowJo package (TreeStar).

### Statistical analysis

Results are expressed as means ± SEM or IQ (as appropriate). Multiple group comparisons were performed by the analysis of variance test. One-way ANOVA was used to compare one variable with at least three groups as parametric test followed by Šídák's multiple comparisons test. Two-way ANOVA was used to compare more than two variables with at least three groups followed by Tukey's multiple comparison test. Changes in microbiological rate infection was compared using Kruskall-Wallis test as non-parametric test followed by Dunn's multiple comparison test. All statistical analyses were done with the GraphPad Prism software (GraphPad Software, LLC, v.10.1.2). Unsupervised principal component analysis with clinical, microbiological and laboratory variables described in Table 1 was performed using Stata Statistical Software, Release 16. College Station, TX: StataCorp LLC.

## Results

### Principal component analysis reveals bacterial infection related patterns in stable KTR

**Figure 1:**
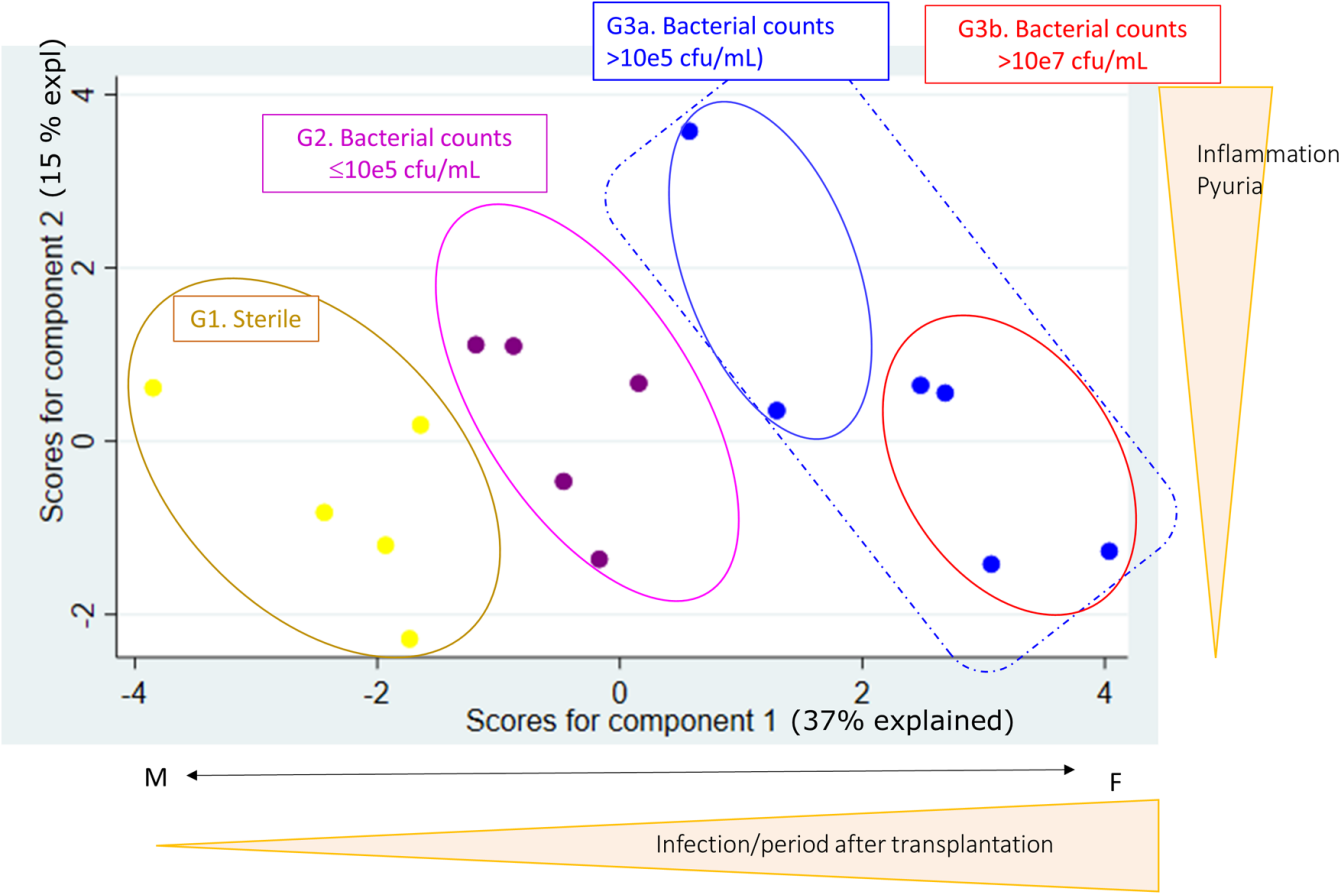
Using principal component analysis (PCA), with clinical, microbiological and laboratory variables summarized in Table 1, four groups were established based on urine analysis. Group 1, denominated sterile, are KTR with no bacteria counts (yellow dots). Group 2 are KTR with gram-negative counts ≤10^5^ cfu/ml (violet dots). Group 3 are patients with gram-negative counts >10^5^ cfu/ml, of which Group 3b is a subgroup of patients with high-grade bacteria counts >10^7^ cfu/ml, 3 of 4 patients were treated with antibiotics and 1 of 4 developed pyelonephritis (blue dots).

We included 16 stable patients with available clinical and microbiological information in our analysis. Table 1 summarises the characteristics of the patients. As shown in Figure 1, a principal component analysis (PCA) was performed with all variables listed in Table 1 and four groups were observed. Groups 1, 2, 3a and 3b are discriminated by component 1 that includes the variables related to infection. It is important to note that all patients in group 3 (3a and 3b), consisting of patients with bacterial infection, had no symptoms of dysuria, fever or chills at the time of urine sample collection. None of the patients in group 3a were treated with antibiotics. However, in group 3b clinical evaluation advised antibiotic treatment in 3 patients due to high pathogen concentration. Another particular case in this group was a patient with no clinical manifestations of UTI at the time of urine sample collection, who 24 h later consulted emergency and was diagnosed with pyelonephritis. These 4 patients were grouped as the 4th group (named group 3b).

### The CD141/CD1c/DC-SIGN phenotype can be found in the urine sample of patients associated with high bacterial infection

Urine samples are known to be characterised by low cell counts. To assess whether a sufficient number of cells could be obtained, samples were centrifuged, cells were counted and live cell counts were assessed by flow cytometry. As shown in Supplementary Material Figure S1A, although the cell concentration shows a large variation per ml of urine, no significant differences were observed between the four groups of patients and we were able to obtain a sufficient number of cells for flow cytometry analysis. It is important to note that when samples come from patients with non-inflammatory conditions, such as the group 1, larger volumes of urine were required, as shown in Supplementary Material Figure S1B. Live cell counts after exclusion of dead cells and Lin^+^ cells (CD3—T cells, CD19—B cells, CD56—NK cells, and CD66b—neutrophils) were at a median of 8115 (2025–18372 IQR) (Supplementary Material Figure S2C). These results provide evidence that a sufficient number of live cells can be obtained in urine samples for downstream analysis of HLA-DR cells, representing 1.5% of the total cells (+/- 0.27 SEM) and 13.41% of the live cells (+/- 3.05 SEM) (Supplementary Material Table S1).

**Figure 2:**
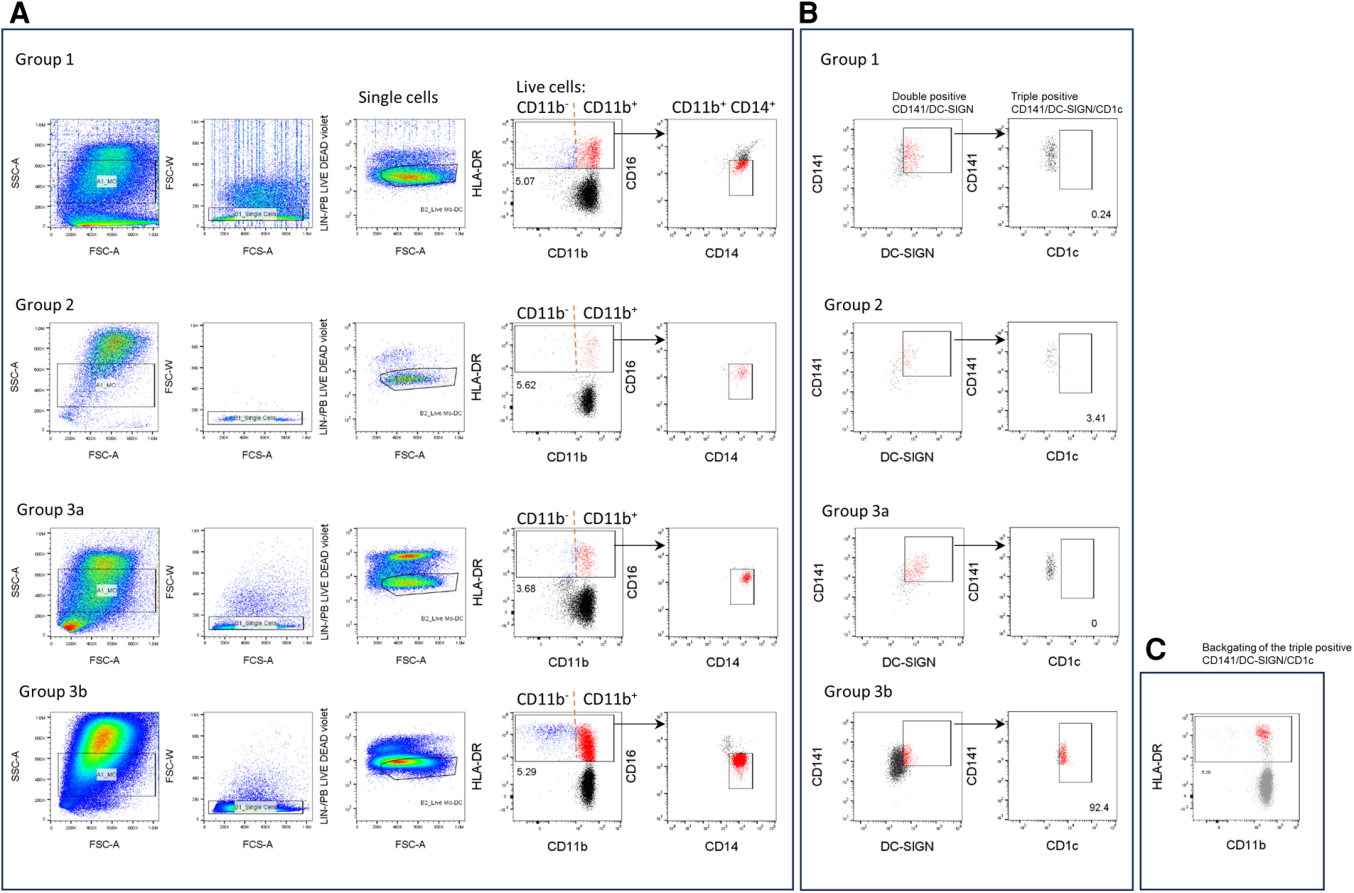
Phenotypic analysis of the CD141/CD1c/DC-SIGN population in urine samples. Cells were stained with anti-lineage (Lin) (CD3, CD19, CD56, CD66b), HLA-DR, CD11b, CD14, CD16, CD141, CD1c and DC-SIGN antibodies and analysed by flow cytometry. (**A**, **B**) One representative experiment is shown for each group of patients. (**A**) From the FSC/SSC gate, cells were gated into singles, lin^−^ and live cells. Then cells were gated by HLA-DR expression and further selected by CD11b expression. Subsequent analysis of the triple positive CD141/CD1c/DC-SIGN population was performed on CD11b + population and CD14^+^ and CD16^−^ cells (red). (**B**) Dot plots of the double positive CD141/DC-SIGN population (red) and further analysis of the CD1c population (red). (**C**) Triple positive ModDC population (red) is backgated onto HLA-DR /CD11b plot (gray).

To determine whether ModDC can be found in urine, cells were stained using a combination of phenotypic markers found on monocytes and DCs. Figures 2A–C show representative dot plots of each KTR group classified according to the PCA. Upon the exclusion of debris, doublets, Lin^+^ cells and dead cells, live cells were gated by HLA-DR expression and further selected by their CD11b expression with the aim of differentiating classical DCs from monocytes. Cells were then further selected for CD14^+^ and CD16^−^ expression (Figure 2A). Figure 2B illustrates the CD141^+^/DC-SIGN^+^ double population and the further selection of the CD1c^+^ population. High frequencies of this triple positive population can be found in the 3b group of patients with high bacterial infection. This population was backgated on the HLA-DR plot. It's high HLA-DR expression level within the CD11b/CD14 population can be clearly observed, which associates them with ModDCs (Figure 2C).

**Figure 3:**
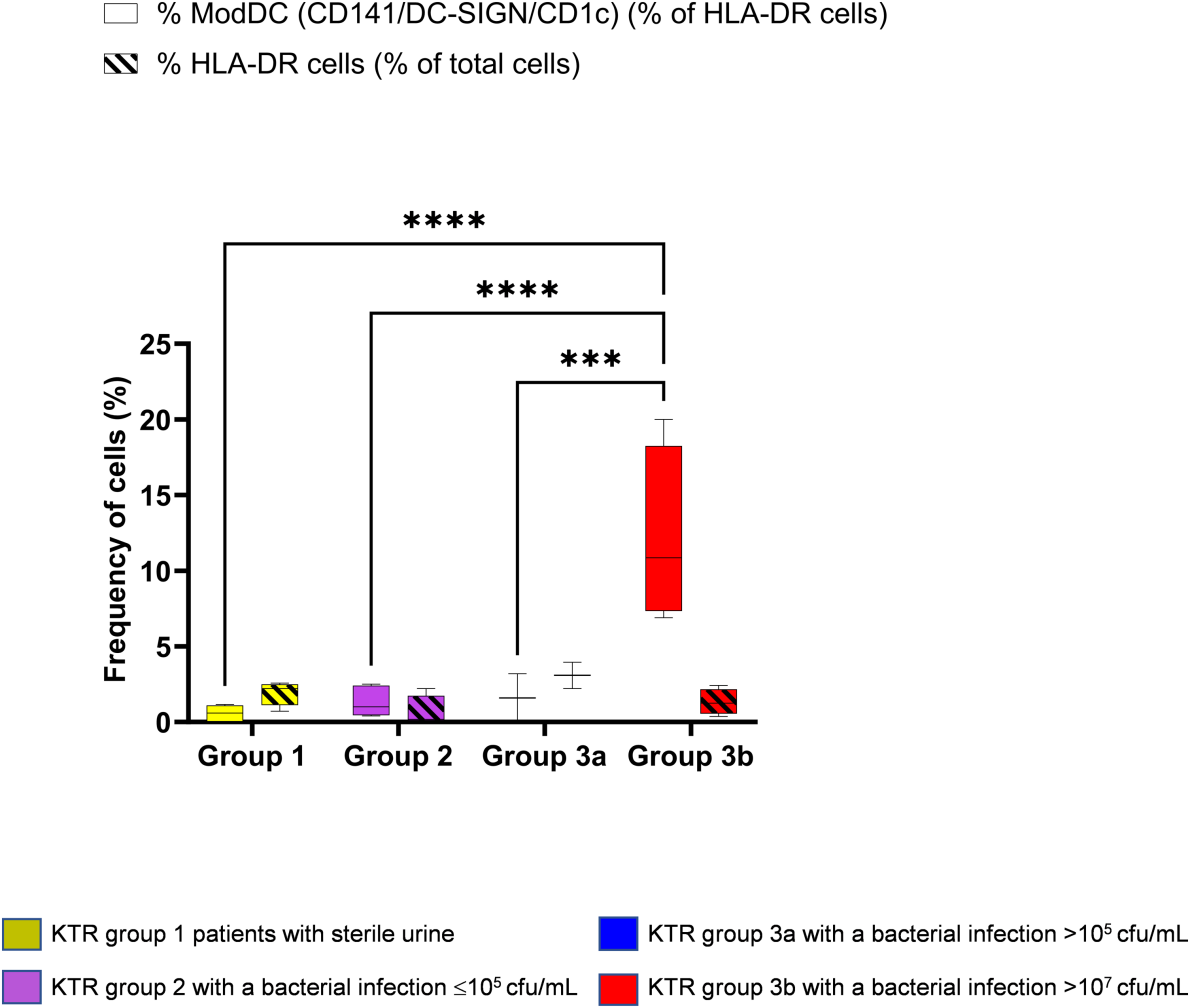
Identification of the modDC triple positive phenotype (CD141 ^+ ^DC-SIGN ^+ ^CD1c^+^) in KTR. Using principal component analysis (PCA), four groups were established as mentioned in Figure 1 and gated as mentioned in Figure 2. Percentage of the triple positive CD141/CD1c/DC-SIGN population amongst HLA-DR ^+ ^CD11b^+^ and CD14^+^/CD16^−^ (open box). Percentage of HLA-DR population among total cells (diagonal line pattern). Data are depicted in box-and-whisker graph. Multiple group comparisons were made by the analysis of variance test (ANOVA). The analysis was followed by Šídák's multiple comparisons test (****P < 0.0001) (***P < 0.001).

Figure 3 depicts the frequency of the CD141/CD1c/DC-SIGN ModDC on the CD14 ^+ ^CD11b^+^ population as well as the frequency of HLA-DR population of total cells in urine samples. While the frequency of HLA-DR population remained stable in all patients with a median of 1.4% (0.4575–2.370) of total cells (diagonal line pattern box), the frequency of ModDCs among the HLA-DR population (open box) was significantly increased in group 3b with a median of 10.8% (7.350–18.25), the group exhibiting a high bacterial load, although no explicit clinical symptoms of infection were present at the time of urine sample collection. Of note, the number of ModDC, although not significant, increased in this group 3b, suggesting that this phenotype is not only increasing in frequency among HLA-DR cells, but may also be more abundant in absolute numbers (Supplementary Material Table S1). The fact that one patient developed pyelonephritis in the following days, could suggest that the frequency of this phenotype in the urine would reflect the severity of infection and may precede clinical evidence. As the three other patients were treated with antibiotics, the potential clinical outcome without treatment is unknown. This result contrasts with the low frequency of this phenotype in patient group 3a, none of whom were treated with antibiotics. This observation opens the hypothesis that among patients with ASB the local immune system may respond differently.

## Discussion

To our knowledge, the present study provides for the first time evidence that ModDCs can be detected in urine from patients with local inflammation due to bacterial infection. Although leukocyte analysis in urine poses significant limitations, especially in conditions with minimal inflammation, multiparameter flow cytometry analysis is emerging as a valuable tool for characterising different cellular phenotypes, including rare populations (22). Although the HLA-DR marker is widely used in the gating strategy for the characterization of monocytes, macrophages and DCs, specific reports on the proportion of HLA-DR cells among the total cells are scarce. HLA-DR population in blood represent 11% of total cells (23), however in bronchoalveolar lavage they only reached 0.85% (+/- 0.8 SEM) (unpublished data). The current results show that the HLA-DR population represents 1.4% of the total cells and 13.41% of the live cells in urine allowing ModDCs analysis. Although this percentage is lower than in blood, it is higher than in other fluids. Furthermore, recent studies have shown that the frequency of monocytes/macrophages in the urine of lupus nephritis patients is 10 times higher than that of T cells, highlighting the importance of analysing HLA-DR cells in this fluid (22, 24).

The relevance of being able to assess this cellular subset in urine comes from the fact that it has been demonstrated that ModDCs are at least one of the migratory populations capable of inducing Th2 polarisation in axillary lymph nodes (23). Furthermore, as reported by Segura et al, tissue ModDCs are able to cross-present antigens and stimulate CD8 effector T cells in inflamed tissues. It is thus essential to conduct thorough research to determine whether ModDCs cell frequency systematically increases during ASB or UTI and is involved in kidney graft dysfunction or in the initiation of acute rejection (25, 26).

From a clinical point of view, the identification of non-invasive biomarkers to discriminate pathogenic ASB from that which can be resolved by the immune system is relevant in the context of renal transplantation. As mentioned above, both ASB and UTI episodes are frequent in KTR, but if ASB cannot be resolved by the immune system or does not progress to UTI with clear clinical symptoms, it represents a risky event for transplant recipients where the delay in the infection diagnosis may impact the allograft function and survival. What is noteworthy is that the difference between these two outcomes, depends on the presence of local or systemic symptoms, which in KTR is arguable since immunosuppressants hold back the inflammatory immune response. Additionally, the kidney is denervated, which could minimize pain symptoms in the context of pyelonephritis. This is probably one of the reasons why KTR with ASB or even low-grade bacterial infection can end up with the rapid development of pyelonephritis or graft rejection without early explicit symptoms before the acute event (5, 27).

This study has a very small number of patients and describes a specific event without the context of infection frequency rate, antibiotic resistance or comorbidities. However, we observed a significant increase in the frequency of ModDCs in the urine of a group of patients with no local or systemic symptoms. Of these, one patient not treated with antibiotics then developed pyelonephritis, thus highlighting the possible involvement of ModDCs as local immune effectors during bacterial infection. Clinical studies should integrate the cellular markers shown in the present study to better discriminate pathogenic from self-resolving ASB.

This preliminary evidence corroborates the existence of ModDCs *in vivo* and the feasibility of monitoring this DC population by flow cytometry. These results highlight not only the involvement of ModDCs during acute inflammation, but also the diagnostic value of urine samples. A large-scale prospective study involving transplant and non-transplant patients with clearly defined ASB and UTI outcomes is needed to better characterize the DCs and ModDCs populations. This will help to better understand local and systemic immune activation following ASB and UTI and determine the subsequent immunogenicity towards an allograft of pathogen-activated DC subsets.

## Data Availability

The raw data supporting the conclusions of this article will be made available by the authors, without undue reservation.

## Supplementary material

Supplementary Figure S1 (**A**) Cell number expressed as cell concentration/mL (yellow circle) and total number of cells counted in urine samples (black square) grouped according to PCA classification. Results are expressed as means ± range. (**B**) Histogram represent the total volume (mL) of urine samples in each KTR group. C: Histogram represent numbers of live cells. Cells were stained LIVE/DEAD® Fixable Dead Cell Stain. Results are expressed as means ± SEM. Data was not significant.
