# Supplementary material for: Monocyte-derived dendritic cells can be detected in urine of kidney transplant recipients with pathogenic asymptomatic bacteriuria
Source: Front Transplant. 2024 Jun 12;3:1366104. doi: 10.3389/frtra.2024.1366104 (PMC11235355; doi:10.3389/frtra.2024.1366104)
Supplement: Supplementary file 1 [file Datasheet1.docx]

**Supplementary Table S1.** Frequencies and counts of ModDC and HLA-DR populations grouped according to PCA analysis.

Flow cytometry analysis

| Variables | Group 1.  Sterile  (n=5) | | Group 2.  Bacterial counts ≤10^5^cfu/mL  (n=5) | | Group 3a  Bacterial counts >10^5^cfu/mL  (n=2) | | Group 3b  Bacterial counts >10^7^cfu/mL  (n=4) | | *p* |
| --- | --- | --- | --- | --- | --- | --- | --- | --- | --- |
| ModDC (% of HLA-DR), mean (SEM) | 0.49 | (0.21) | 1.28 | (0.45) | 1.58 | (1.58) | 12.08 | (2.82) | **<0.0001**^a^ |
| ModDC (absolute number), mean (SEM) | 6.6 | (4.42) | 10.2 | (7.27) | 59.4 | (73) | 182 | (96) | 0.097 ^a^ |
| HLA-DR (% of total cells), mean (SEM) | 1.89 | (0.34) | 0.77 | (0.42) | 2.28 | (1.61) | 1.3 | (0.6) | 0.303 ^a^ |
| HLA-DR (% of live cells), mean (SEM) | 15.2 | (0.5) | 9.02 | (3.39) | 24.74 | (21.06) | 11.0 | (4.3) | 0.493^a^ |

SEM: standard error of the mean. ModDC, monocyte-derived dendritic cells. ^a^ One-way ANOVA.
