# Supplementary figures and images for: Monocyte-derived dendritic cells can be detected in urine of kidney transplant recipients with pathogenic asymptomatic bacteriuria
Source: Front Transplant. 2024 Jun 12;3:1366104. doi: 10.3389/frtra.2024.1366104 (PMC11235355; doi:10.3389/frtra.2024.1366104)

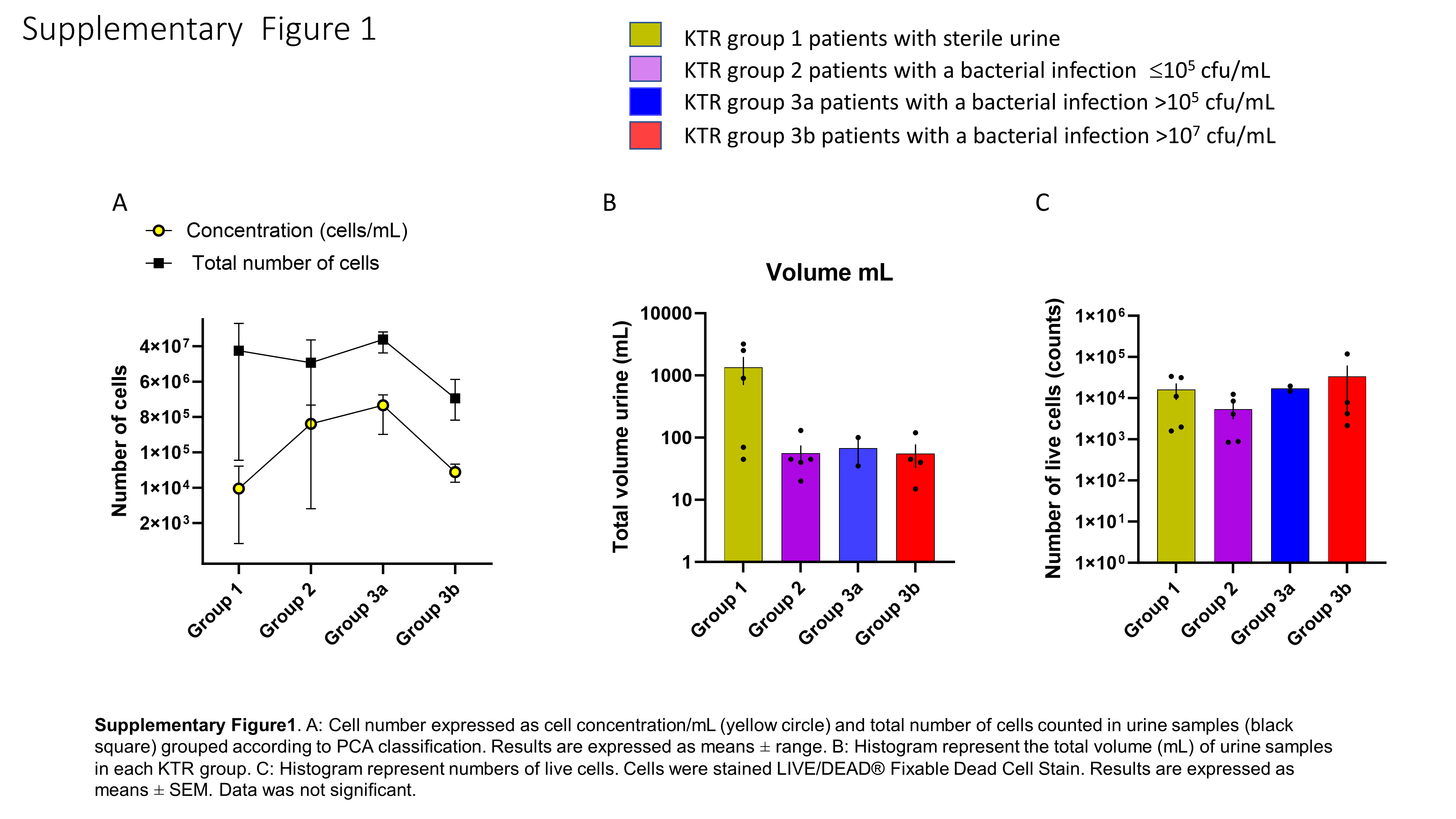

Supplement: Supplementary Figure S1 — (A) Cell number expressed as cell concentration/mL (yellow circle) and total number of cells counted in urine samples (black square) grouped according to PCA classification. Results are expressed as means ± range. (B) Histogram represent the total volume (mL) of urine samples in each KTR group. C: Histogram represent numbers of live cells. Cells were stained LIVE/DEAD® Fixable Dead Cell Stain. Results are expressed as means ± SEM. Data was not significant. [file Image1.tif]
